# Perinatal depression screening and prevention: Descriptive findings from a multicentric program in the South of Italy

**DOI:** 10.3389/fpsyt.2022.962948

**Published:** 2022-08-05

**Authors:** Antonello Bellomo, Melania Severo, Annamaria Petito, Luigi Nappi, Salvatore Iuso, Mario Altamura, Alessia Marconcini, Elisa Giannaccari, Giuseppe Maruotti, Giuseppe Luigi Palma, Mario Vicino, Antonio Perrone, Anna Maria Tufariello, Valeria Sannicandro, Eleonora Milano, Giulia Arcidiacono, Melanie Di Salvatore, Antonella Caroli, Isabella Di Pinto, Antonio Ventriglio

**Affiliations:** ^1^Department of Clinical and Experimental Medicine, University of Foggia, Foggia, Italy; ^2^Department of Medical and Surgical Sciences, University of Foggia, Foggia, Italy; ^3^Unit of Gynecology, Di Venere Hospital, Bari, Italy; ^4^Unit of Gynecology, Vito Fazzi Hospital, Lecce, Italy; ^5^Unit of Psychology, Vito Fazzi Hospital, Lecce, Italy; ^6^Dipartimento Promozione Salute, Regione Puglia, Bari, Italy; ^7^Dipartimento Promozione Della Salute e del Benessere Animale, Regione Puglia, Bari, Italy

**Keywords:** perinatal depression, pregnancy, EPDS, screening, prevention

## Abstract

**Introduction:**

Perinatal depression (PD) is a cluster of clinical depressive symptoms occurring globally during pregnancy or after childbirth, with a prevalence of 11.9%. Risk factors for PD among pregnant women may include personality traits of neuroticism, low personal resilience, higher anxiety, avoidance in close relationships, as well as dysfunctional coping strategies.

**Methods:**

We report on descriptive findings of a screening/prevention program aimed to detect depressive symptoms and associated risk factors in a large sample of women (*N* = 1,664) accessing the gynecological departments of the Regione Puglia (South of Italy) from July to November 2020. Pregnant women were assessed in their third trimester of pregnancy (T0), after childbirth (T1), and those at risk for PD within 1 year from delivery (T2–T4); The Edinburgh Postnatal Depression Scale (EPDS) has been employed for the screening of PD over time as well as other standardized measures for neuroticism, resilience, coping strategies, and quality of life.

**Results:**

Of 1,664, *n* = 1,541 were tested at T1, and 131 scored ≥ 12 at EPDS (14.6 ± 2.95), showing a higher risk for PD. They were followed over time at 1, 6, and 12 months after childbirth (T2–T4), and 15 of them scored ≥ 12 (EPDS) at T4. Women with a higher risk of PD also reported higher levels of neuroticism, lower levels of personal resilience, more anxiety and avoidance in close relationships, higher employment of dysfunctional coping strategies (e.g., denial, self-blame, etc.), and lower quality of life (0.0008 < *all p* < 0.0001).

**Conclusion:**

This study confirmed the benefit of screening programs for the early detection of PD among pregnant women. We may suggest a set of risk factors to be considered in the clinical assessment of PD risk as well as the promotion of similar programs to improve depressive outcomes and pathways to care for PD on the basis of a more accurate assessment and referral.

## Introduction

It has been largely recognized that motherhood and pregnancy lead to a set of significant adjustments for the woman in terms of bodily changes, psychological adjustment (including the awareness of becoming a mother), and changes in relationships (1–4). Also, the hormonal and physiological changes occurring with the pregnancy are unique and may impact the neurobiological functioning of the mother’s brain circuits (5). Postpartum depression (PPD) is a complex mood disorder occurring within 4 weeks to 1 year after the delivery, characterized by physical, emotional, and behavioral changes that happen after giving birth (6). Recently, Putnick et al. (7) reported that depressive symptoms have occurred up to 3 years after delivery among 4,866 pregnant women from the general population. Clinical characteristics of postpartum depressive episodes may include psychotic features, suicidal behaviors, and child murder (8, 9). The prevalence of PPD in the general female population is 17% (10); its clinical diagnosis includes the criteria for the Major Depressive Episode (MDE), according to the DSM-5 [Diagnostic and Statistical Manual of Mental Disorders: Fifth edition (11)], with the specifier of “*Postpartum Onset*” used if the onset of MDE occurs in close proximity (within 4 weeks) to childbirth.

Perinatal Depression (PD) refers to a cluster of clinical depressive symptoms occurring globally during pregnancy or after childbirth, with a prevalence of 11.9% (12). PD risk factors among pregnant women include low level of education, lower socioeconomic status, poorly supportive partner, low social support, a higher number of stressful life events, and previous history of depression and anxiety (12). Yang et al. (13) reported the personality trait of *neuroticism* as an additional risk factor for PD, as it may lead to psychological vulnerability to stressful events, sleep deprivation, and hormonal changes (14, 15): neuroticism has been described as an independent risk factor for PD, and many authors suggest the psychological assessment of this factor in the clinical screening of pregnant women (16). The Edinburgh Postnatal Depression Scale (EPDS) by Cox et al. (17) is a validated tool for the clinical detection of PD and depressive symptoms during pregnancy (see description in the “Materials and methods” section). The *insecure attachment* is also considered to be a psychological factor associated with a higher risk of PD since it is related to *internal working models* based on a negative self-representation (18, 19). Finally, the subjective vulnerability to develop PD is associated with dysfunctional *coping* strategies as well as protective factors for PD, which are related to personal psychological factors of *resilience* (20, 21).

All these pieces of evidence suggest an accurate assessment of risk factors for depression and vulnerability among pregnant women to early detect depressive symptoms and prevent PD (22–27). Stephens et al. (28) suggested that early detection of depressive symptoms in the peripartum and following early intervention with appropriate treatments, where needed, may significantly improve mothers’ depressive outcomes in the mid-long term with benefits on children’s development. These pieces of evidence encourage screening programs and early intervention for PD in the general pregnant population (29).

We here report on descriptive findings of a large program of screening and prevention, aimed to early detect clinical features and psychological and socio-cultural risk factors for PD among pregnant women accessing the gynecological departments of the Regione Puglia in the south of Italy, from July to November 2020 and followed-up from their third trimester of pregnancy to 1 year after the childbirth.

## Materials and methods

### Sample and study design

This study reports on descriptive findings of a screening/prevention program aimed to detect depressive symptoms and associated risk factors in a large sample of women accessing the gynecological departments of the Regione Puglia (South of Italy). A total of 1,664 women were recruited in their third trimester of pregnancy and followed up in the *peripartum*, considered the period between childbirth and 1 year after the delivery. The enrollment involved pregnant women accessing the Units of Gynecology of Policlinico Riuniti di Foggia (Foggia, Italy), Ospedale Vito Fazi di Lecce (Lecce, Italy), and Ospedale Di Venere di Bari (Bari, Italy). A total of 1,664 women in their third trimester of pregnancy were enrolled from July to November 2020. The assessment included standardized and validated psychological tools exploring mothers’ personality traits, depressive and anxious symptoms, and a set of risk factors associated with PD as suggested by the international literature. The first step of assessment (T0) was set at 15–45 days before the delivery; mothers were screened employing EPDS at T0 and within the seventh day after childbirth (T1). For those women reporting significant levels of depressive symptoms as well as risk factors at the T1 step, multistep monitoring was programmed at 1 month (T2), 6 months (T3), and 1 year after the delivery (T4) with a re-testing on the EPDS. All participants provided their written informed consent, including their agreement on privacy and anonymous data processing. The exclusion criteria included poor language proficiency (Italian), intellectual disability, and women aged < 18 years old. The program involved well-trained psychiatrists, one statistician, psychiatric trainees, and psychologists from each center of the multicentric study (Foggia, Bari, Lecce).

### Aim

This project aimed to develop an accurate screening—a program of risk factors for PD among women accessing the Gynecology Units of the largest hospitals in the Regione Puglia from July to November 2020. Also, early detection and referral of women with higher risk for PD were programmed as well as the assessment of their psychosocial characteristics.

### Supportive psychological intervention and psychoeducation

A set of psychoeducational sessions was provided during the hospitalization (T0) in the gynecological units on the following topics: parenting, difficulties after childbirth, such as breastfeeding or infant crying, concerns about body shape and sexuality, social support, and mother’s role-adjustment required (30). In addition, information on physiological as well as non-physiological emotions reported in the postpartum (e.g., differences between *baby blues* and PPD) were provided. These sessions were carried out by well-trained psychologists in perinatal psychology and psychopathology. Specific psychological support was also provided through empathic listening, encouragement to express negative feelings, recognition of maternal ambivalence, and normalization of destabilizing emotional experiences (31, 32). Women reporting a PD-risk profile (EPDS score ≥ 12) were informed of their own condition and referred to *ad hoc* territorial services (e.g., family counseling services and psychological services). Also, women with a higher risk of PD, as well as reporting with a history of psychiatric disorders, familiarity with mood disorders, and/or previous psychotherapy and pharmacotherapy, were referred to the local Mental Health Centers (Foggia, Bari, Lecce).

Table 1 reports the detailed study time schedule and steps (T0, T1, T2, T3, and T4).

**TABLE 1:** Study time-schedule and steps (T0-T4).

Time schedule		Training (investigators)	T0 (45–15 days before the CB)	T1 (7 days from CB)	T2 (1 month from CB)	T3 (6 months from CB)	T4 (12 months from CB)	Package 2	Package 3	Package 4	Package 5	Statistical analyses	Themed conferences and congresses
*Year Month*			Package 1	EPDS	EPDS	EPDS	EPDS						
2020	June	x											X
	July		X	X				X	X	X			X
	August		X	X	X			X	X	X			
	September		X	X	X			X	X	X		X	
	October		X	X	X			X	X	X			X
	November			X	X			X	X	X		X	
	December				X					X			
2021	January					X				X	X		
	February					X				X	X		X
	March					X				X	X		
	April					X				X	X		
	May					X				X	X		
	June					X				X	X		
	July						X			X			
	August						X			X			
	September						X			X		X	X
	October						X			X			X
	November						X			X		X	X

PD, Perinatal Depression; CB, childbirth; Package 1: EPDS, The Edinburgh Postnatal Depression Scale; NEO-FFI, The N scale of the 60 items NEO Five-Factor Inventory; ECR-S, The Experience in Close Relationship Scale; CD—RISC, The Connor-Davidson Resilience Scale; The Brief—COPE; The WHOQOL BREF, World Health Organization Quality of Life; Package 2: Supportive Psychological Intervention and Psychoeducation; Package 3: risk assessment and supportive intervention planning for women with mid-high risk of PD; Package 4: patients referral (ambulatories and PD dedicated units); Package 5: follow-up of women with no/low risk of PD.

### Assessment

Each participant was interviewed for the collection of personal sociodemographic variables, information on personal medical history (including the history of psychological distress or mental disorders), and social support. The following standardized and validated tools were employed:

- EPDS (17): It is widely employed and validated for the screening of PD in different sociocultural settings. It was proposed by Cox and Holden in 1987 and describes PD as depressive symptoms within the last 7 days of observation, including 10 items rated on a 4-point Likert scale ranging from 0 to 3, with a total score ranging from 0 to 30. A total score of ≥ 12 is considered as significantly associated with the clinical risk of PD. This tool reports a high level of sensibility and specificity in different cultures. EPDS is internationally recognized as a valid screening test for PD as recommended by the National Institute for Health and Care Excellence guidelines [NICE; (17, 33)]. It has been translated into many languages and validated for assessment during pregnancy and postpartum. We employed the Italian version by Benvenuti et al. (34).

The N scale of the 60 items NEO Five-Factor Inventory (NEO-FFI) by McCrae and Costa (35): This tool explores Neuroticism (N), Extraversion (E), and Openness (O). We employed the N subscale based on 12 items rated on a 5-point Likert scale ranging from 0 to 4, describing Neuroticism, defined as a fundamental personality trait associated with a higher level of anxiety, depression, or negative experiences (worry, fear, anger, frustration, loneliness, etc.). Higher scores report higher levels of neuroticism. The mean score in healthy adult women was reported to be around 16.77 ± 7.91 (35). As already discussed, this personality trait was considered among personality-related risk factors for PD (13–16).

The Experience in Close Relationship (ECR) by Brennan et al. (36): It is a self-administered tool composed of 36 items describing the attachment style to the partner as well as assessing individual differences with respect to attachment-related anxiety (the extent to which people are insecure vs. secure about the availability and responsiveness of a romantic partner) and avoidance (the extent to which people are uncomfortable in being close to others vs. secure depending on others). Higher scores report higher levels of anxiety or avoidance. The questionnaire shows a high level of validity and internal consistency. We employed the Italian version by Picardi et al. (37).

The Connor-Davidson Resilience Scale (CD—RISC) by Connor and Davidson (38): It describes resilience as the ability to withstand adversity and bounce back from difficult life events. The tool includes 25 items exploring resilience within the last month. Patients’ answers may rate as follows: not true at all (0), rarely true (1), sometimes true (2), often true (3), and true nearly all of the time (4). These ratings result in a number between 0 and 100; higher scores indicate higher levels of resilience. Domains explored are personal competence, high standards, and tenacity; trust in one’s instincts, tolerance of negative affect, and strengthening effects of stress; positive acceptance of change and secure relationships; control; and spiritual influences. The CD-RISC has been reported to have good convergent validity.

The *Brief—*COPE by Carver (39): It is a 28-item self-report questionnaire designed to measure effective and ineffective ways to cope with a stressful life event. “Coping” is defined broadly as an effort used to minimize distress associated with negative life experiences. The following facets are described: active coping, items 2 and 7 (Problem-Focused); use of informational support, items 10 and 23 (Problem-Focused); positive reframing, items 12 and 17 (Problem-Focused); planning, items 14 and 25 (Problem-Focused); emotional support, items 5 and 15 (Emotion-Focused); venting, items 9 and 21 (Emotion-Focused); humor, items 18 and 28 (Emotion-Focused); acceptance, items 20 and 24 (Emotion-Focused); religion, items 22 and 27 (Emotion-Focused); self-blame, items 13 and 26 (Emotion-Focused); self-distraction, items 1 and 19 (Avoidant); denial, items 3 and 8 (Avoidant); substance use, items 4 and 11 (Avoidant); and behavioral disengagement, items 6 and 16 (Avoidant). Each of the 14 scales consists of 2 items; thus, total scores on each scale range from 2 to 8. Higher scores indicate greater use of one specific coping strategy. We employed the Italian version as provided by Conti (40).

The WHOQOL BREF by de Girolamo et al. (41): It is a quality of life (QOL) assessment developed by the World Health Organization WHOQOL Group in 1996 (42). It explores the quality of life as an individual’s perception of his/her position in life in the context of the culture and value system in which he/she lives and in relation to goals, expectations, standards, and concerns. Twenty-six items explore the following domains: physical health; psychological health; social relationships; and environment.

### Ethical approval

This program was designed by the Unit of Psychiatry at University of Foggia in cooperation with the Units of Gynecology of Policlinico Riuniti di Foggia/University of Foggia (Foggia, Italy), Ospedale Vito Fazi di Lecce (Lecce, Italy), and Ospedale Di Venere di Bari (Bari, Italy). It was proposed and approved by the Regione Puglia with two special deliberations: “DGR n. 1392 released on 2 August 2018 and DGR n. 2294 released on 11 December 2018.” This project is a part of the plan promoted by the Department of Health Promotion of the Regione Puglia entitled “*Governo dell’assistenza alle persone in condizione di fragilitaÌ,”* approved with a special deliberation n. 65 released on 12 March 2019.

Ethical approvals were obtained by each local ethical committee: Policlinico Riuniti di Foggia/University of Foggia (Foggia, Italy), Ospedale Vito Fazi di Lecce (Lecce, Italy), and Ospedale Di Venere di Bari (Bari, Italy). All participants provided written informed consent, and participation was free of any charge. Data and information were treated with confidentiality, equality, and justice, respecting the Helsinki principles.

### Statistical analyses

Analyses were conducted using the statistical software Grand Prism 5 (San Diego, CA, United States). Means and standard deviations (SDs) as well as% rates were calculated for each characteristic and parameter, and findings were considered statistically significant with a two-tailed *p-value* ≤ 0.05. The differences in characteristics across the groups were compared using the non-parametric Kruskal–Wallis test with Dunn’s multiple comparison *post hoc* testing (our sample has been enrolled by a large distribution-free population). The assessments of the relationship between psychological characteristics and EPDS scores were performed using Pearson’s correlation (*r*).

## Results

A sample of 1,664 women, aged 32.4 ± 5.50 years old, was enrolled in the third trimester of pregnancy. A total of 79% of women accessing the Units of Gynecology from July to November 2020 agreed to join the study. Their sociodemographic and clinical characteristics at intake (T0) are reported in Table 2.

**TABLE 2:** Socio- demographic and clinical characteristics at intake (T0; *N* = 1664).

Characteristics		Means ± *SD* or *n*/%-rate (*N* = 1664)
**Current age (*years old*)**		32.4 ± 5.50
Education, *n* (%)*	Primary	11 (0.66%)
	Secondary	174 (10.4%)
	Post-secondary	834 (50.2%)
	Higher-education	640 (38.5%)
**Marital status,** *n* (%)**	Single	16 (0.96%)
	Widower	0 (0.00%)
	Separated	5 (0.30%)
	Divorced	2 (0.12%)
	Married	1,104 (66.3%)
	Engaged	481 (28.9%)
	Coupled	55 (3.31%)
**Employment,** *n* (%)***	Yes	924 (55.9%)
	No	729 (44.1%)
**Previous psychological or mental disorders (6 months), *n* (%)**	No	1,377 (82.7%)
	Mood disorders	40 (2.40%)
	Eating disorders	22 (1.32%)
	Substance abuse	0 (0.00%)
	Anxiety disorder	176 (10.5%)
	Alcohol abuse	0 (0.00%)
	Psychosis	0 (0.00%)
	Other	49 (2.94%)
**Gynecological condition, *n* (%)**	Primigravida	936 (56%)
	Previous spontaneous abortions	473 (28%)
	Premenstrual syndrome	826 (50.9%)
Complications in pregnancy, *n* (%)****	No	1,166 (70.3%)
	Abortion threat	92 (5.55%)
	Hypertension	14 (0.84%)
	Gestational diabetes	145 (8.75%)
	Blood loss	72 (4.34%)
	Leakage of amniotic fluid	6 (0.36%)
	Other	104 (6.27%)
	≥ 2 Complications	59 (36.5%)

*5 missing subjects/N = 1,664; **1 missing subject/N = 1,664; ***11 missing subjects/N = 1,664; ****6 missing subjects/N = 1,664.

Education level among participants were rated as 11 (0.66%) with primary education; 174 (10.4%) with secondary education; 834 (50.2%) with post-secondary education; and 640 (38.5%) with higher education. The majority of the participants who reported to being currently employed was 924 (55.9%). Self-reported previous psychiatric disorders in the 6 months preceding the pregnancy were as follows: 40 (2.40%) with mood disorders; 22 (1.32%) with eating disorders; 0 (0.00%) with substance abuse; 176 (10.5%) with anxiety disorder; 0 (0.00%) with alcohol abuse; 49 (2.94%) with other disorders; and 1,377 (82.7%) with no disorders. The psychological assessment and its characteristics at the intake (T0) are described in Table 3.

**TABLE 3:** Psychological assessment at intake (T0; *N* = 1,664).

Characteristics		Means ± *SD* or %-rate (*N* = 1,664)
**EPDS**	T0: ≥ 12, *n* (%)	6.46 ± 4.49 235 (14)
	T1: ≥ 12, *n* (%)	131 (7.87) 14.6 ± 2.95
**NEO**		14.7 ± 7.31
**ECR**	Anxiety	41.9 ± 18.7
	Avoidance	29.9 ± 13.6
**CD-RISC**		78.2 ± 13.6
**Brief—COPE**	Positive reinterpretation and growth	6.14 ± 1.47
	Self-distraction	5.27 ± 1.68
	Focus on and venting of emotions	4.93 ± 2.03
	Use of informational support	5.37 ± 1.60
	Active coping	6.66 ± 1.39
	Denial	3.24 ± 1.43
	Religion	4.91 ± 2.01
	Humor	4.25 ± 1.37
	Behavioral disengagement	2.93 ± 1.26
	Emotional support	4.94 ± 1.65
	Substance use	2.08 ± 0.52
	Acceptance	6.24 ± 1.35
	Planning	6.40 ± 1.46
	Self-blame	5.06 ± 1.47
**WHOQOL BREF**	Psychological wellbeing	12.3 ± 2.48
	Social relations	12.1 ± 1.83

EPDS, The Edinburgh Postnatal Depression Scale; NEO-FFI, The N scale of the 60 items NEO Five-Factor Inventory; ECR-S, The Experience in Close Relationship Scale; CD—RISC, The Connor- Davidson Resilience Scale; The Brief—COPE; The WHOQOL BREF, World Health Organization Quality of Life.

The general mean score on EPDS in the sample was 6.46 ± 4.49, reporting a general level of depressive symptoms below the significant cutoff. Splitting the sample at T0 on the base of distribution over and below the clinical cutoff of 12, we found 235 (14%) women reporting a significant level of depressive symptoms with a high risk of PD (EPDS ≥ 12) and 1,429 (86%) reporting no significant depressive symptoms and low risk of PD (EPDS < 12). Women reporting a relevant risk of PD at T1 (EPDS ≥ 12) were 131 (7.87%; mean score 14.6 ± 2.95).

Neuroticism at NEO Five-Factor Inventory, as an associated risk factor for PD, scored 14.7 ± 7.31, which is considered a low level of neuroticism in this general population. Anxiety and avoidance in close relationships assessed by ECR scored 41.9 ± 18.7 and 29.9 ± 13.6, respectively, confirming low levels. Protective factors to PD, measured at CD-RISC assessment for personal resilience, reported a total score of 78.2 ± 13.6, describing a medium-to-low level of resilience. Coping strategies were described on Brief-COPE as follows: a tendency to use adaptive coping strategies such as positive reinterpretation and growth (6.14 ± 1.47), self-distraction (5.27 ± 1.68), use of instrumental support (5.37 ± 1.60), active coping (6.66 ± 1.39), acceptance (6.24 ± 1.35), and planning (6.40 ± 1.46). There is a tendency to employ maladaptive coping strategies, such as self-blame (5.06 ± 1.47). Finally, the quality of life of participants was evaluated at WHOQOL BREF, reporting total sub-scores of 12.3 ± 2.48 and 12.1 ± 1.83, describing psychological wellbeing and quality of social relationships in the normal range.

Of 1,664, 1,541 were retested within 7 days after delivery (T1) using EPDS. Of these, 131 (9%; mean age 33.3 ± 5.20 years old) reported a significant level of depressive symptoms with a relevant risk of PD (EPDS ≥ 12) and 1,410 (91%) reported no significant depressive symptoms and risk of PD (EPDS < 12). In total, *n* = 59/131 women at PD-risk after delivery (T1) were tested to be already at risk (EPDS ≥ 12) at T0. The project included the follow-up (1 month, 6 months, and 1 year) after delivery of those women reporting a PD confirmed risk at T1: their sociodemographic and clinical characteristics are reported in Table 4.

**TABLE 4:** Socio- demographic and clinical characteristics at intake for women reporting EDPS total score ≥ 12 (T1; *N* = 131).

Characteristics		Means ± *SD* or *n*/%-rate (*N* = 131)
**Current age (*years old*)**		33.3 ± 5.20
Education, *n* (%)*	Primary	0 (0.00)
	Secondary	21 (16.0)
	Post-secondary	67 (51.1)
	Higher-education	42 (32.0)
**Marital status, *n* (%)**	Single	1 (0.76)
	Widower	0 (0.00)
	Separated	0 (0.00)
	Divorced	0 (0.00)
	Married	90 (68.7)
	Engaged	36 (27.4)
	Coupled	4 (3.05)
**Employment,** *n* (%)**	Yes	78 (59.5)
	No	51 (38.9)
**Previous psychological or mental disorders (6 months), *n* (%)**	No	87 (66.4)
	Mood disorders	14 (10.6)
	Eating disorders	4 (3.05)
	Substance abuse	0 (0.00)
	Anxiety disorder	26 (19.8)
	Alcohol abuse	0 (0.00)
	Psychosis	0 (0.00)
	Other	0 (0.00)
**Gynecological condition, *n* (%)**	Primigravida	64 (48.8)
	Previous spontaneous abortions	40 (30.5)
	Premenstrual syndrome	91 (69.4)
Complications in pregnancy, *n* (%)***	No	88 (67.1)
	Abortion threat	7 (5.34)
	Hypertension	1 (0.76)
	Gestational diabetes	15 (11.4)
	Blood loss	7 (5.34)
	Leakage of amniotic fluid	1 (0.76)
	Other	9 (6.87)
	≥ 2 Complications	2 (1.52)

*1 missing subjects/N = 131; **2 missing subject/N = 131; ***1 missing subjects/N = 131.

Education level among participants was rated as follows: 0 (0.00%) with primary education;, 21 (16%) with secondary education; 67 (51.1%) with post-secondary education; and 42 (32.0%) with higher education. The majority of the participants who reported to be currently employed was 78 (59.5%). Self-reported previous psychiatric disorders in the 6 months preceding the pregnancy were as follows: 14 (10.6%) with mood disorders; 4 (3.05%) with eating disorders; 0 (0.00%) with substance abuse; 26 (19.8%) with anxiety disorder; 0 (0.00%) with alcohol abuse; 0 (0.00%) with other disorders; and 87 (66.4%) with no disorders. The psychological assessment and its characteristics after childbirth (T1) are described in Table 5.

**TABLE 5:** Psychological assessment at intake for women reporting EPDS total score ≥ 12 (T1; *N* = 131).

Characteristics		Means ± *SD* (*N* = 131)
**EPDS**		14.6 ± 2.95
**NEO**		21.2 ± 9.05
**ECR**	Anxiety	57.2 ± 23.0
	Avoidance	37.1 ± 16.6
**CD-RISC**		69.9 ± 16.0
**Brief—COPE**	Positive reinterpretation and growth	5.96 ± 1.58
	Self-distraction	5.54 ± 1.58
	Focus on and venting of emotions	5.37 ± 1.45
	Use of informational support	5.65 ± 1.57
	Active coping	6.44 ± 1.44
	Denial	3.80 ± 1.57
	Religion	5.00 ± 1.93
	Humor	3.93 ± 1.47
	Behavioral disengagement	3.33 ± 1.40
	Emotional support	5.63 ± 1.73
	Substance use	2.17 ± 0.63
	Acceptance	5.96 ± 1.41
	Planning	6.40 ± 1.36
	Self-blame	5.66 ± 1.57
**WHOQOL BREF**	Psychological wellbeing	10.6 ± 2.74
	Social relations	11.3 ± 1.89

EPDS, The Edinburgh Postnatal Depression Scale; NEO-FFI, The N scale of the 60 items NEO Five-Factor Inventory; ECR-S, The Experience in Close Relationship Scale; CD—RISC, The Connor- Davidson Resilience Scale; The Brief—COPE; The WHOQOL BREF, World Health Organization Quality of Life.

The general mean score on EPDS in this sub-group was 14.6 ± 2.95, reporting a significant overall level of depressive symptoms and PD risk.

Neuroticism at NEO Five-Factor Inventory, as an associated risk factor for PD, scored 21.2 ± 9.05, which is considered a high level of neuroticism compared with the mean score of healthy adult women. Anxiety and avoidance in close relationships, as assessed by the ECR, scored low: 57.2 ± 23.0 and 37.1 ± 16.6, respectively. CD-RISC assessment for personal resilience reported a total score of 69.9 ± 16.0, describing a medium-low level of resilience. Coping strategies were described on Brief-COPE as follows: a tendency to use adaptive coping strategies such as positive reinterpretation and growth (5.96 ± 1.58), self-distraction (5.54 ± 1.58), use of instrumental support (5.65 ± 1.57), active coping (6.44 ± 1.44), acceptance (5.96 ± 1.41), and planning (6.40 ± 1.36). There was a tendency to employ maladaptive coping strategies, such as self-blame (5.66 ± 1.57). Finally, the quality of life of participants was evaluated at WHOQOL BREF, reporting total sub-scores of 10.6 ± 2.74 and 11.3 ± 1.89, describing psychological wellbeing and quality of social relationships in a normal range.

**FIGURE 1:**
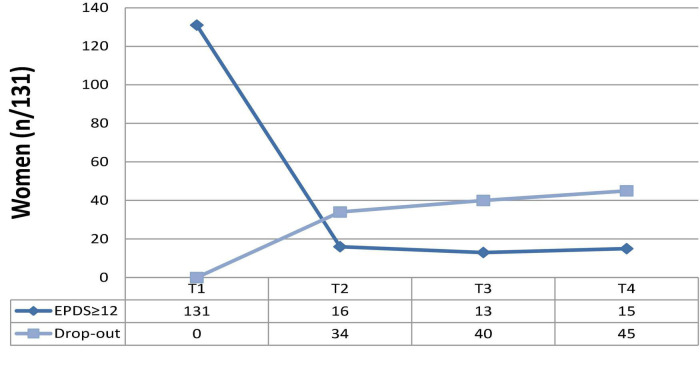
Re-test and drop-out rates of women reporting EPDS total scores ≥ 12 at T1 (screening; *N* = 131).

Of the 131 women at risk for PD, 31% (*n* = 41) accepted to be referred to *ad hoc* services. At T2, 16/131 (12.2%) reported an EPDS score of ≥ 12 and 34/131 (25.9%) dropped out. At T3, 13/131 (9.92%) scored ≥ 12 on EPDS and 40/131 (30.5%) dropped out. At T4, 15 (11.4%) scored significantly and 45 (34.3%) dropped out (Table 6 and Figure 1).

**TABLE 6:** Re-test and drop-out rates of women reporting EPDS total scores ≥ 12 at T1 (screening; *N* = 131).

Study time-line	Women with EPDS ≥ 12; *N* = 131	
	EPDS ≥ 12 *n/131 (%)*	Drop-out *n/131 (%)*
**T1**	131 (100)	-
*(Referral)* *	41 (31.0)	90 (69)
**T2**	16 (12.2)	34 (25.9)
**T3**	13 (9.92)	40 (30.5)
**T4**	15 (11.4)	45 (34.3)

*31% (n = 41) accepted to be referred to ad hoc services after the screening.

At the final follow-up (T4), 15/131 women reported significant depressive symptoms (11.4%), whereas the rest of the sample showed a decrease in symptoms, including women referred to *ad hoc* services [31% (*n* = 41)]. The final rate of drop-out was 34.3% (*n* = 45), due to the personal unavailability of women to be retested during the study steps (T2, T3, T4, as described).

### Psychological characteristics among women at higher risk of perinatal depression

Table 7 shows the psychological characteristics measured for women at T1 (*n* = 1,541, tested at 7 days after the delivery); a comparison was made between women testing at EPDS < 12 (*n* = 1,410; *3 incomplete data available* = 1,407) vs. EPDS ≥ 12 (*n* = 131). We found significantly lower scores of neuroticism (NEO), anxiety, and avoidance (Experience in Close Relationship Scale) among women not at risk of PD (*n* = 1,410; EPDS<12; all *p* < 0.0001). Also, women not at risk of PD at T1 reported higher levels of resilience (CD-Risk; *p* < 0.0001), lower levels of focus and venting of emotions, denial, behavioral disengagement, use of emotional support, self-blame (BRIEF-Cope; 0.0008 < *all p* < 0.0001), as well as higher levels of humor (BRIEF-Cope; *p* = 0.0049) and higher levels of quality of life in general (WHOQOL BREF; all *p* < 0.0001).

**TABLE 7:** Comparison of psychological characteristics between women reporting EPDS scores < 12 (*n* = 1,407) *or* ≥ 12 (*n* = 131) at T1.

Characteristics		EPDS < 12 (*n* = 1,407)	EPDS ≥ 12 (*n* = 131)	*P*-value
		Means ± *SD*		
**EPDS**		4.75 ± 2.91	14.6 ± 2.95	<0.0001
**NEO**		14.1 ± 0.18	21.2 ± 9.05	<0.0001
**ECR**	Anxiety	40.8 ± 0.46	57.2 ± 23.0	<0.0001
	Avoidance	29.2 ± 0.34	37.1 ± 16.6	<0.0001
**CD-RISC**		78.9 ± 0.34	69.9 ± 16.0	<0.0001
**Brief—COPE**	Positive reinterpretation and growth	6.17 ± 1.46	5.96 ± 1.58	0.1261
	Self-distraction	5.36 ± 1.60	5.54 ± 1.58	0.0555
	Focus on and venting of emotions	4.90 ± 0.04	5.37 ± 1.45	0.0008
	Use of informational support	5.34 ± 0.04	5.65 ± 1.57	0.0342
	Active coping	6.78 ± 1.32	6.44 ± 1.44	0.0589
	Denial	3.18 ± 0.03	3.80 ± 1.57	<0.0001
	Religion	4.76 ± 2.03	5.00 ± 1.93	0.6425
	Humor	4.29 ± 1.35	3.93 ± 1.47	0.0049
	Behavioral disengagement	2.87 ± 1.22	3.33 ± 1.40	0.0002
	Emotional support	4.89 ± 0.04	5.63 ± 1.73	<0.0001
	Substance use	2.06 ± 0.47	2.17 ± 0.63	0.0241
	Acceptance	5.96 ± 0.12	5.96 ± 1.41	0.0200
	Planning	6.52 ± 1.41	6.40 ± 1.36	0.8723
	Self-blame	5.02 ± 0.03	5.66 ± 1.57	<0.0001
**WHOQOL BREF**	Psychological well-being	12.5 ± 2.39	10.6 ± 2.74	<0.0001
	Social relations	12.1 ± 0.04	11.3 ± 1.89	<0.0001

EPDS, The Edinburgh Postnatal Depression Scale; NEO-FFI, The N scale of the 60 items NEO Five-Factor Inventory; ECR-S, The Experience in Close Relationship Scale; CD—RISC, The Connor- Davidson Resilience Scale; The Brief—COPE; The WHOQOL BREF, World Health Organization Quality of Life.

Finally, we tested the correlations between the psychological characteristics and total EPDS scores in the whole sample at T1 (*n* = 1,541) and found that higher scores at EPDS are positively correlated to higher levels of neuroticism (NEO), anxiety, and avoidance in the close relationships (ECR), self-distraction, focus and venting of emotions, use of informational support, behavioral disengagement, use of emotional support, and self-blame (all at Brief-COPE). Higher scores at EPDS are significantly correlated to lower levels of resilience (CD-Risk), humor, positive reinterpretation and growth, acceptance (all at Brief-COPE), as well as lower levels of quality of life (WHOQOL BREF) (Table 8).

**TABLE 8:** Correlations (Pearson’s *r*) between psychological characteristics and EPDS total score among women assessed at T1 (*n* = 1,541).

Characteristics		EPDS at T1 (*n* = 1,541)	*P*-value
		*r*	
**NEO**		0.3984	<0.001
**ECR**	Anxiety	0.3412	<0.001
	Avoidance	0.2057	<0.001
**CD-RISC**		–0.2667	<0.001
**Brief—COPE**	Positive reinterpretation and growth	–0.05949	<0.05
	Self-distraction	0.040	<0.001
	Focus on and venting of emotions	0.1086	<0.001
	Use of informational support	0.07770	<0.01
	Active coping	–0.022	0.0130
	Denial	0.2039	<0.001
	Religion	0.026	0.0428
	Humor	–0.027	0.0018
	Behavioral disengagement	0.064	<0.001
	Emotional support	0.1503	<0.001
	Substance use	0.005	0.1209
	Acceptance	–0.08200	<0.01
	Planning	–0.016	0.0827
	Self-blame	0.1802	<0.001
**WHOQOL BREF**	Psychological wellbeing	–0.171	<0.001
	Social relations	–0.2132	<0.001

EPDS, The Edinburgh Postnatal Depression Scale; NEO-FFI, The N scale of the 60 items NEO Five-Factor Inventory; ECR-S, The Experience in Close Relationship Scale; CD—RISC, The Connor- Davidson Resilience Scale; The Brief—COPE; The WHOQOL BREF, World Health Organization Quality of Life.

## Discussion and conclusion

Many authors have reported that pregnancy and childbirth may be stressful events leading to mental health issues for women, above all for those affected by previous mental disorders, in particular severe mood disorders (43–45). Thus, the pregnant women population should be routinely and accurately screened for risk factors and symptoms of depression (29).

In this study, we report on descriptive findings of a large screening/prevention program aimed to detect depressive symptoms and associated risk factors in a large sample of women accessing the gynecological departments of the Regione Puglia (South of Italy). *N* = 1,664 women were recruited in their third trimester of pregnancy and followed-up in the peripartum, considered as the period between childbirth and 1 year after the delivery. Also, psychosocial predictive factors of PD have been assessed and personalized pathways to care have been indicated for those women at higher risk of PD (referral).

Of the 1,664 women recruited at their third trimester of pregnancy, 1,541 were retested within 7 days after delivery (T1) using EPDS, with 131 (9%; mean age 33.3 ± 5.20) reporting a significant level of depressive symptoms with a risk of PD (EPDS ≥ 12). We employed the EPDS as one of the most recommended tools in the scientific literature for accurate screening of depressive symptoms in the peripartum (46, 47). In fact, it has been efficaciously employed and approved in different settings such as primary care and gynecological units (47). According to the international literature (48), the EDPS cut-off ≥ 12, considered in this study, has been associated with a higher risk of PD, as confirmed by the following clinical assessment of those women reporting positive scores. In addition, we described and measured the socio-demographic and psychological characteristics of those women with a higher risk of PD. The percentage of women at risk for PD was lower at T1 (7 days after childbirth) than at T0 (9 vs. 14%) and, in general, lower than prevalence findings reported in the international literature: Yin et al. (49) have shown a percentage of risk for PD around 20%, whereas Shorey et al. have detected 17% of PPD among 37.294 women (10). Notably, the percentage of PD risk in our sample refers to the scoring of EPDS ≥ 12 and is not considered as a proper clinical diagnosis of PD. We found that a higher risk of PD was associated with the report of previous mental disorders. In particular, among 131 women with PD-risk at T1, *n* = 14 (10.6%) have suffered from mood disorders and *n* = 26 (19.8%) from anxiety disorders. This evidence has been widely confirmed in the literature (11, 50). In our sample, *n* = 41/131 (31%) at higher risk of PD accepted to be referred to an *ad hoc* service and *n* = 31 of them (76%) reported a significant reduction of symptoms of EPDs at T4. The final drop-out rate (at T4, 1 year of follow-up after the childbirth) was 34.5% (*n* = 45/131), mostly due to the personal unavailability of women to be retested during the study steps. This finding has been previously discussed in the literature: similar screening programs reported that the rates of discontinuation were high, with more than 30% of women missing the follow-up appointments (51, 52), even if the benefits of these screening and prevention programs have been largely recognized (53). For instance, a controlled trial based on personal psychotherapy for 120 women affected by PPD has shown a discontinuation rate of more than 50% with 20% of complete dropout among participants (54). These pieces of evidence do not discourage these screening/prevention programs but suggest developing strategies for improving adherence among the pregnant women population (54). In fact, 59 out of 131 (45%) women at higher risk for PD at T1 have reported higher scores in the previous assessment (T0) as well as a previous history of mental disorders; this confirms the evidence from the literature that symptoms of depression and anxiety during pregnancy as well as preexisting mental disorders are significant risk factors for PD and need to be followed-up over time (50).

In terms of prevention, our study has not been specifically designed for measuring the impact of psychoeducational sessions provided during the hospitalization (T0) in the gynecological units (psychoeducation on parenting, difficulties after childbirth, such as breastfeeding or infant crying, concerns about body shape and sexuality, social support, and mother’s role-adjustment required). The reduction of PD-risk women at T1 vs. T0 (131 vs. 235) may be partially due to the effect of this intervention, even if any specific evaluation has been made. However, this program successfully detected PD-risk subjects in a large sample of pregnant women and allowed themselves to be promptly referred to *ad hoc* services as part of the secondary prevention process.

We found higher levels of neuroticism among *n* = 131 at risk for PD than in the rest of the sample (*n* = 1,407), with a significant positive correlation between neuroticism levels and depressive symptoms; this may strongly confirm that neuroticism is a specific risk factor for PD as suggested by the literature (55–57). It is of interest that some authors consider neuroticism as a psychological *endophenotype* of affective disorders (58) as well as a personality characteristic independently leading to PD with adjunctive vulnerability to stressful life events, sleep disorders, and hormonal changes (13–15). These pieces of evidence suggested the inclusion of neuroticism assessment in our screening program (16). Also, lower levels of resilience (CD-RISC) have been confirmed among our PD-risk women (*n* = 131) when compared with the rest of the sample (*n* = 1,407); women scoring ≥ 12 on the EPDS reported lower levels of personal resilience than the other at-risk patients tested by Connor and Davidson (38). Also, Lubián López et al. reported that, during the COVID-19 pandemic’s first peak, the personal resilience levels of pregnant women were negatively correlated with the severity of mood and anxiety symptoms (59). Lower levels of resilience seem to play as mediators between trait-anger and PPD among pregnant women (60) as well as between stress and anxiety symptoms during pregnancy and PPD (61). These findings suggest that interventions aimed to increase women’s personal resilience may be protective in terms of the prevention of PD (21).

Levels of anxiety and avoidance in close relationships (ECR) have been assessed with higher scores among PD-risk women in our sample (*n* = 131). Pieces of evidence from the literature suggest that higher levels of anxiety and avoidance are associated with higher scores at EPDS (62). Since the transition phase to motherhood may be stressful for many women, those with an insecure attachment style may develop affective disorders after childbirth with the activation of internal working models based on a negative self-representation (18, 63). Zhang et al. (64) showed that primigravida women with an insecure attachment reported a higher prevalence of PD with lower emotional bonding between mother and child.

Prevalent coping strategies, as assessed among PD-risk women of our sample (*n* = 131), were denial, emotional support, and self-blame. Also, women with a high risk of PD (EPDS ≥ 12) reported more self-distraction, lower levels of focus and venting of emotions, lower use of informational support, denial, behavioral disengagement, use of emotional support, and self-blame as well as lower levels of acceptance and positive reinterpretation and growth (at Brief-COPE). These findings confirm that all dysfunctional coping strategies are associated with higher levels of depressive symptoms and may be risk factors for PD. In fact, some authors reported that the employment of coping strategies, such as self-distraction, substance use, and self-blame among 1,626 (observed in 32 weeks), have been predictive factors of PPD (65, 66). Gutiérrez-Zotes et al. (67) suggested that maladaptive strategies such as denial, distancing, self-blame, and substance abuse have been associated with higher levels of PD symptoms as well as a higher probability to develop PPD. Conversely, positive reinterpretation and growth seem to be protective of mood disorders (as confirmed in our sample with a negative correlation between these specific scores and general score at EPDS). Also, women with depressive symptoms show more avoidant behaviors than those with no levels of depression (68). A change of the coping strategies through psychoeducational programs is one of the aims of the therapeutic approaches for reducing vulnerability to stress in patients at risk of depression after childbirth (69). In fact, psychological interventions based on cognitive behavioral techniques are encouraged for improving dysfunctional coping strategies (69, 70). The quality of life assessed in the sample was rated differently among at-risk vs. not-at-risk women, with those reporting higher scores on EPDS having shown lower levels of quality of life at WHOQOL BREF, as confirmed in the literature (71, 72).

Finally, it is of note that premenstrual syndrome, affecting 69.4% of our sample (91/131), is considered among risk factors for mood disorders in pregnant women (73). Further analyses might be conducted to describe the role of this specific characteristic in the depressive outcome of pregnant women.

Limitations of this study may include a higher number of dropout probably due to the difficulties in accessing the hospital units in the course of the COVID-19 pandemic: this trend has been largely described in different clinical settings (74). Moreover, future studies should assess the impact of the COVID-19 pandemic on women’s health; in fact, recent pieces of evidence suggested that pregnant women during the pandemic were at higher risk of developing psychopathological issues and that PPD rates have been increasing probably due to the adjunctive stress related to social distancing and fear of COVID-19 (75). Also, the timing of study follow-up (e.g., re-test at 1 and 6 months after the childbirth; Table 1) may have impacted the rate of dropout for the following reasons: participants may have lost motivation over time (5 months) and women with depressive symptoms may have asked for help outside this protocol. However, all participants have been provided with full details on how to contact our team in case of urgent need and active listening was provided within the length of all studies when required/needed. In addition, clinical characterization of PD-risk women should be detailed, and more information on their specific treatments might be collected. Further analyses might also describe the role of the identified risk/protection factors in the clinical outcome of PD-risk patients. Another limitation may include the lack of specific information regarding the outcome of PD-risk women (*n* = 41) referred to specialized services; Six of them were regularly followed up and treated at Policlinico di Foggia with a cognitive-behavioral approach and a successful control of symptoms over time; diagnostic and outcome information regarding the rest of sub-group (*n* = 35) followed in external services is not available at the moment and may be collected in the second step of this study.

We here conclude that many pieces of evidence encourage programs of screening and early intervention for PD and other mental disorders in the general pregnant population. This study reports on descriptive findings of a large screening/prevention program aimed to detect depressive symptoms and associated risk factors in a large sample of women accessing the gynecological departments of Regione Puglia (South of Italy). The results may suggest a set of risk factors to be considered in the clinical assessment of PD risk, such as neuroticism, lower personal resilience, higher anxiety, and avoidance in close relationships as well as personal dysfunctional coping strategies. Also, we suggest promoting further prevention and screening programs to early detect PD and properly address mental health problems of women with a specific PD risk profile. These initiatives may also improve outcomes and pathways to care for PD on the basis of a more accurate assessment and referral.

## Data availability statement

The raw data supporting the conclusions of this article will be made available by the authors, without undue reservation.

## Ethics statement

The studies have been proposed and approved by the Regione Puglia with two special deliberations “DGR n. 1392 released on 2 August 2018 and DGR n. 2294 released on 11 December 2018.” This project is part of the plan promoted by the Department of Health Promotion of the Regione Puglia entitled “Governo dell’assistenza alle persone in condizione di fragilitaÌ” approved with a special deliberation n. 65 released on 12 March 2019. The patients/participants provided their written informed consent to participate in this study.

## Author contributions

AB, MS, APet, and AV wrote the manuscript. All authors joined the project actively and contributed to the article and approved the submitted version.
